# The Melding of Drug Screening Platforms for Melanoma

**DOI:** 10.3389/fonc.2019.00512

**Published:** 2019-06-24

**Authors:** Gabriela Klein Couto, Natália Vieira Segatto, Thaís Larré Oliveira, Fabiana Kömmling Seixas, Kyle M. Schachtschneider, Tiago Collares

**Affiliations:** ^1^Research Group in Molecular and Cellular Oncology, Postgraduate Program in Biochemistry and Bioprospecting, Cancer Biotechnology Laboratory, Center for Technological Development, Federal University of Pelotas, Pelotas, Brazil; ^2^Biotechnology Graduate Program, Molecular and Cellular Oncology Research Group, Laboratory of Cancer Biotechnology, Technology Development Center, Federal University of Pelotas, Pelotas, Brazil; ^3^Department of Radiology, University of Illinois at Chicago, Chicago, IL, United States; ^4^Department of Biochemistry & Molecular Genetics, University of Illinois at Chicago, Chicago, IL, United States; ^5^National Center for Supercomputing Applications, University of Illinois at Urbana-Champaign, Urbana, IL, United States

**Keywords:** drug screening, melanoma, *in silico*, *in vitro*, *in vivo*, cancer, 3R, B16 melanoma

## Abstract

The global incidence of cancer is rising rapidly and continues to be one of the leading causes of death in the world. Melanoma deserves special attention since it represents one of the fastest growing types of cancer, with advanced metastatic forms presenting high mortality rates due to the development of drug resistance. The aim of this review is to evaluate how the screening of drugs and compounds for melanoma has been performed over the last seven decades. Thus, we performed literature searches to identify melanoma drug screening methods commonly used by research groups during this timeframe. *In vitro* and *in vivo* tests are essential for the development of new drugs; however, incorporation of *in silico* analyses increases the possibility of finding more suitable candidates for subsequent tests. *In silico* techniques, such as molecular docking, represent an important and necessary first step in the screening process. However, these techniques have not been widely used by research groups to date. Our research has shown that the vast majority of research groups still perform *in vitro* and *in vivo* tests, with emphasis on the use of *in vitro* enzymatic tests on melanoma cell lines such as SKMEL and *in vivo* tests using the B16 mouse model. We believe that the union of these three approaches (*in silico, in vitro*, and *in vivo*) is essential for improving the discovery and development of new molecules with potential antimelanoma action. This workflow would provide greater confidence and safety for preclinical trials, which will translate to more successful clinical trials and improve the translatability of new melanoma treatments into clinical practice while minimizing the unnecessary use of laboratory animals under the principles of the 3R's.

## Introduction

The global incidence of cancer is rapidly rising and remains a leading cause of death worldwide (1), highlighting the need for ongoing research focused on the discovery and development of new drug candidate molecules as well as new treatments. According to the World Health Organization (WHO), about 8.8 million people die of cancer each year (2). With regard to Brazil, around 600,000 new cancer cases are expected for the biennium 2018–2019 (3). This is partially due to the increased incidence of Melanoma in recent years (4). Not only does melanoma represents one of the fastest growing forms of cancer, but its advanced metastatic forms carry high mortality rates due to their development of resistance to drugs traditionally used to treat melanoma (5).

In order to determine the optimal treatment strategy, melanoma patients must be evaluated and classified into stages. For stages 0 and I, surgery for tumor excision is generally the preferred treatment option. For stage II, or stage I with positive sentinel lymph node biopsy, adjuvant treatment with interferon is preferred. For stage III where the tumor has already metastasized to the lymph nodes, surgery with wide excision in addition to adjuvant treatment with interferon is preferred. If the patient does not respond, the remaining options include: bacillus Calmette-Guérin (BCG) immunotherapy, interleukin-2 (intralesional), radiotherapy, imiquimod application, and chemotherapy (see Table 1 for a list of the most commonly used chemotherapeutics for melanoma treatment). Stage IV melanomas are especially difficult to treat. Chemotherapy with dacarbazine and temozolomide may be used individually or in combination with interleukin-2 and/or interferon. In recent years, therapies such as immunotherapy and targeted therapies have proven to be more effective than the traditional chemotherapy (21). Although early-stage melanoma can be treated with surgery, advanced (metastatic) disease is difficult to cure and treatment options are unsatisfactory, highlighting the urgent need for novel treatment strategies (22).

**Table 1:** Chemotherapeutics most commonly used for treatment of melanoma.

	***In vitro*** **tests**			***In vivo*** **tests**							
**chemotherapy**	**Institute**	**Cell line**	**Human blood**	**Mouse/camundongos**	**Rabbit**	**Dogs /monkey**	**Monkey**	**Clinical tests**	**Approval date^a^**	**Company**	**References**
Dacarbazine^d^ (also called DTIC)	-American Cancer Society; -Brazilian Ministry of Health; - National Cancer Institute.	Information not found.	Analysis of human peripheral blood lymphocytes	Carcinogenicity	Risk for Pregnancy	Information not found.	Information not found.	PHASE II and III	May.27.1975/ FDA	BAYER HLTHCARE	(6–9)
Temozolomide^d^	-American Cancer Society; -Brazilian Ministry of Health.	Information not found.	Clastogenic analysis in human lymphocytes	– Carcinogenicity; –Toxicology profile.	Toxicology profile.	–Testicular atrophy was observed; –Toxicology studies were performed.	Information not found.	PHASE III and IV	Aug.11.1999/ FDA	MERCK SHARP DOHME	(9, 10)
Nab-paclitaxel^b^	American Cancer Society.	CHO cell line –mutagenicity test	clastogenic analysis in human lymphocytes.	– Genotoxicity; –Risk for pregnancy.	Information not found.	Information not found.	Information not found.	PHASE II and III	Dec. 29, 1992/ FDA	HQ SPCLT PHARMA	(11–13)
Cisplatin^c^	-American Cancer Society; -Brazilian Ministry of Health.	–Mutagenic test; –Chromosomal abnormalities in cell lines.	Information not found.	–Drug is teratogenic, embryotoxic, carcinogenic and leukemogenic; –Regression of tumors in mice was observed.	Information not found.	Information not found.	Information not found.	PHASE III	Dec. 19, 1978 /FDA	HQ SPCLT PHARMA	(14)
Carboplatin^d^	American Cancer Society.	Genotoxicity assessment	Information not found.	–Evaluation of the lethal dose; –Investigation of toxic effects; –Risk for pregnancy.	Information not found.	–A lethal dose was evaluated; –Investigation of toxic effects.	Information not found.	PHASE II and III	March 3, 1989 /FDA	Uninformed	(15–17)
Vinblastine^d^	American Cancer Society	–Mutagenicity; –There is no information on clastogenicity.	Information not found.	–Risk of Mutagenicity; –There is no information on clastogenicity; –Degenerative changes were observed in germ cells, in animal studies.	Information not found.	Information not found.	Information not found.	PHASE II and III	Nov. 5, 1965/FDA	Uninformed	
Nivolumab^d^	American Cancer Society	*In vitro* assays: -Specific memory response antigen *in vitro*.	Tests carried out: –Mixed lymphocytic reaction; -Stabilization of enterotoxin B by Staphylococcal of PBMCs; -Suppression assay with regulatory T cells	Transgenic mice were immunized for antibody-screening test			- Pharmacokinetics, toxicity and immunogenicity of nivolumab in cynomolgus monkeys; -Imunization of SK-MEL-3 melanoma cells and surface antigen of hepatitis B virus in cynomolgus monkeys.	PHASE III	Dec. 22, 2014 /FDA	BRISTOL MYERS SQUIBB	(18)
Ipilimumab^d^	American Cancer Society		–To evaluate potential action was tested on human lymphocytes; –Evaluate immunotherapy action.	Risk assessment in pregnancy.	Information not found.	Information not found.	-Evaluation of risk pregnancy; –Post abnormalities cement; –Toxicological tests.	PHASE I, II and III	March 25, 2011/FDA	BRISTOL MYERS SQUIBB	(19, 20)

a *http://drugcentral.org*;

b *https://media.celgene.com/content/uploads/sites/19/Abraxane_Bula_Profissional.pdf*;

c *http://pfizer.com.br/sites/g/files/g10027021/f/product_attachments/PlatamineCS_PS.pdf*;

d *https://dailymed.nlm.nih.gov/dailymed/drugInfo.cfm?setid=f073b58e-56d6-4c8d-a2ce-b37719402d77&audience=consumer*;

*http://www.bccancer.bc.ca/drug-database-site/Drug%20Index/Vinblastine_monograph_1Feb2015.pdf; https://clinicaltrials.gov/ct2/show/NCT00213278*.

For new drugs to be available for commercialization, they must first go through the necessary steps mandated by regulatory agencies such as the Food and Drug Administration (FDA)^1^, namely: Discovery and development; preclinical trials, clinical trials, and FDA review. Research and development of new antimelanoma molecules occurs in several ways. Some research groups develop or acquire software in order to test thousands of molecules, aiming to select the most promising candidates for the next tests (*in vitro*/*in vivo*). This development process is known as “*in silico* test” (23, 24). In addition to predicting safety and toxicity, these tests can predict interactions between molecules and their receptors, saving time and money during the process of drug screening. Other groups choose to test some molecules *in vitro* and then select their candidates for future *in vivo* and *ex vivo* trials. Both of these approaches follow the 3R principle: “reduction, replacement, and refinement” of animal use. In order to adhere to this principle, it is important to continuously review and optimize the way screening of new candidate drugs is performed. In addition, a robust initial screening of these molecules provides strong candidates for subsequent preclinical and clinical testing.

**Figure 1:**
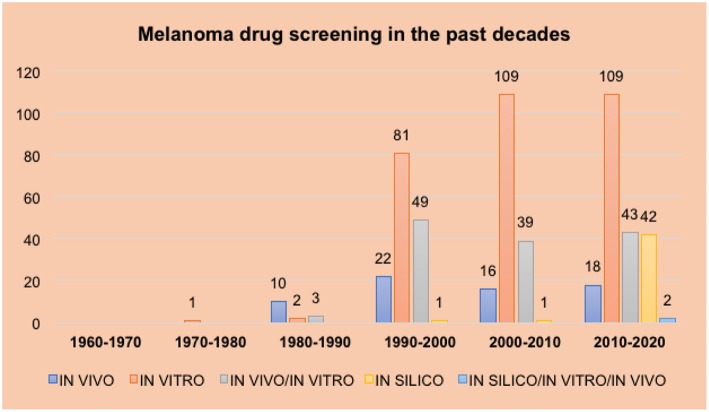
Results indicate the number of articles using each screening methodology by decade. The number of articles found for each topic searched is presented on the y axis. Different decades are presented in the x axis. Each bar represents a different screening method (*in vitro, in vivo*, and *in silico*) and the combination of more than one screening method: dark blue for *in vivo*, orange for *in vitro*, gray for *in vivo/in vitro*, yellow for *in silico* and light blue for all the three screening methods (*in silico/in vitro/in vivo*).

The objective of this review is to analyze the methods used to screen new drug candidate molecules over the last seven decades using articles published during this period (Figure 1). As the use of *in silico* and *ex vivo* methodologies are not as widespread compared with *in vivo* and *in vitro* methodologies, this review is divided into three major sessions according to the chronological order in which these different screening approaches were first utilized.

### Section I

#### *In vitro* Drug Assays for Melanoma

*In vitro* drug screening assays for melanoma are mostly performed to evaluate the cytotoxic potential of new compounds for cancer cell lines and to characterize target mechanisms of action. Several mechanisms have been identified in melanoma regression, including apoptosis pathways, necrosis, and autophagy (25). In addition to cytotoxicity, immune mechanisms are also involved in the therapeutic efficacy against metastatic melanoma, corroborating the use of intralesional BCG as an immunotherapeutic agent (26, 27).

The need to conduct animal research based on 3Rs principle has strengthened the development of novel and more robust *in vitro* models able to better mimic *in vivo* human conditions. Tumor biology is extensively diverse in terms of genetics, pigmentation, morphology, metabolism, and immune microenvironment. A variety of screening techniques have been developed tin an attempt to address this variability. Combination therapies have been clinically employed; however, resistance to therapy has propelled the search for low-cost and rapid screening techniques that allow for selection of new and more effective compounds (28).

In this section, we aim to show the evolution of *in vitro* techniques employed for melanoma drug screening, ranging from conventional assays to novel models for the discovery of more efficient targets.

#### Cell Lines

Most melanoma cell lines used for *in vitro* drug screening are derived from humans. In addition, some studies have explored the use of cells obtained directly from both primary and metastatic tumor biopsies to characterize the potential of novel drugs *in vitro* (29–32). The establishment and analysis of primary melanoma cell cultures is important to investigate tumor heterogeneity in the era of personalized medicine. However, the need to preserve biopsy samples for histological diagnosis may limit their use for *in vitro* drug screening.

Mutations involved in human melanoma progression are commonly observed in *BRAF/NRAS* and *TP53* resulting in altered regulation of the RAS RAF-MEK-ERK and ARF-p53 pathways, respectively (33). As expected, the most commonly used melanoma cell lines harbor many of these mutations (Table 2). In addition, as metastatic melanoma is the most aggressive type of skin cancer, cell lines derived from metastatic tumors (Table 2) are routinely employed to evaluate drugs targeting cell migration and invasiveness (34).

**Table 2:** Human melanoma cell lines most frequently used for *in vitro* drug screening studies.

**Human melanoma cell lines**	Genetic characteristics^*^	Pigmentation^*^
LOX-IMVI^#^	*BRAF* ValGlu (600)	Amelanotic
Malme-3M^#^	*BRAF* ValGlu (600) CDKN2A deletion	Pigmented
SKMEL-2^#^	*NRAS* GlnArg (61) *TP53* GlySer (245)	Amelanotic
SKMEL-5^#^	*BRAF* ValGlu (600)	Amelanotic
SKMEL-28	*BRAF* ValGlu (600) *CDK4* ArgCys (24) *EGFR* ProSer (753) *PTEN* ThrAla (167) *TP53* LeuArg (145)	Amelanotic
UACC-62	*BRAF* ValGlu (600) PTEN insertion; frameshift (248)	^**^
UACC-257	*BRAF* ValGlu (600) CDKN2A deletion	^**^
M14^#^	*BRAF* ValGlu (600) *TP53* GlyGlu (266)	Amelanotic
WM1366	*NRAS* GlnLeu (61)	
A375	*BRAF* ValGlu (600)	Amelanotic
SKMEL-1^#^	*BRAF* ValGlu (600) *CTNNB1* SerCys (33)	Pigmented

* *Data obtained from PubMed, ATCC and ExPASy databases*.

# *Derived from metastatic sites*.

** *Information not described*.

Both pigmented and non-pigmented melanoma cell lines have been used to identify drugs capable of improving therapeutic efficacy and avoiding resistance related to melanin's scavenger ability (35). Amelanotic cell lines, especially A375 and SKMEL-28, have been employed for this purpose (36, 37). Sharma et al. demonstrated improved efficacy of a hypericin-based therapy following depigmentation of melanotic and amelanotic cell lines (UCT Mel-1 and A375, respectively) with a tyrosinase-inhibitor, suggesting the melanogenesis process represents a promising target for treating metastatic melanoma (36).

Murine melanoma is mainly represented by studies using the metastatic B16F10 cell line. The B16F10 cell line retains wild-type copies of *TP53, NRAS*, and *BRAF*, although it does harbor deletions of tumor suppressor genes associated with the INK4a/ARF pathway (38). B16 cell lines are frequently employed to induce tumors in murine models for *in vivo* drug screening, which is discussed in detail below.

#### Conventional Assays

The most common assays used for screening drugs for melanoma treatment, as well as for other cancers, are employed to evaluate enzyme activity. Other techniques have been used to determine mechanisms of cytotoxicity, including membrane damage, DNA synthesis blockade, production of reactive oxygen species (ROS, and drug uptake (39).

Assays primarily based on tetrazolium compounds have been used since the 1980s to determine cell viability. These compounds, such as 3- (4,5-dimethylthiazol-2-yl)−2,5-diphenyl tetrazolium bromide (MTT) and 3-(4,5-dimethylthiazol-2-yl)-5-(3-carboxymethoxyphenyl)-2- (4-sulfophenyl)-2H-tetrazolium (MTS), are used as substrates in colorimetric assays to determine the activity level of mitochondrial enzymes. These techniques are often used as initial steps for novel molecule screening. However, there are several disadvantages associated with tests based on detection of mitochondrial enzymatic activity, including the fact that reprogramming of melanoma cells is frequently accompanied by a metabolic switch that uses glycolysis rather than oxidative phosphorylation for energy production, and that tetrazolium compounds can be reduced by other mechanisms independent of enzymatic catalysis, which can lead to a biased result (40). Thymidine incorporation assays are a direct method to measure DNA synthesis during cell division that have been used extensively since the 80s in studies reporting drug evaluation for melanoma (41). However, assays based on isotype incorporation are reported to be more sensitive and reliable than indirect methods to assess cell viability, such as MTT and clonogenic assays (29).

A more recent study used the activity of an acid phosphatase enzyme to determine melanoma cell viability after treatment with kinase inhibitors (42). Kinase proteins are involved in several process that are deregulated in cancers, including melanoma (43). Using Sk-Mel-28 and Sk-Mel-2 cell lines, which harbor *BRAF* and *NRAS* mutants, respectively, the authors screened 160 compounds of which 20 demonstrated the ability to inhibit growth rates by more than 50%. Among them, fascaplysin, a CDK4 inhibitor, demonstrated the ability to induce apoptosis and inhibit growth using a clonogenic assay. CDK4 deregulation due to a lack of expression of the tumor suppressor p16INK4a is associated with 82% of melanoma metastases (44). These results demonstrate that kinase-targeted screening assays are a promising strategy for identification of novel, targeted therapeutics for metastatic melanoma. Other studies employed for preliminary drug screening for melanoma are based on ATP detection through a luminescent signal produced by the luciferase reaction (45, 46).

The production of melanin by melanocytes occurs through activity of a tyrosinase enzyme in an organelle called melanosome (47). It is well established that melanin can contribute to therapeutic evasion and resistance by acting as a chelator agent (48–50). Tyrosinase-targeted drugs may promote melanocyte depigmentation and consequently improve therapeutic susceptibility (36). Thus, the melanogenesis process has also been explored as a therapeutic target since the 90's (48, 51, 52). *In vitro* techniques to evaluate tyrosinase activity have been employed. Riley *et al*. screened a group of phenolic compounds with side-chain variations for melanogenesis-targeted cytotoxicity (51). The authors evaluated tyrosinase mediated oxidation of phenols to quinones using oximetry and spectrophotometry, in addition to the relation with inhibition of thymidine incorporation as a measure of cell viability. Phenols with lipophilic sidechains demonstrated increased melanocytotoxic potential, highlighting the importance of screening organelle-specific drug targets. More recent works have accessed tyrosinase activity using L-dihydroxyphenylalanine (L-DOPA), a precursor for melanin biosynthesis, as a substrate (48, 53).

Studies from as early as the 80s were already seeking to develop screening techniques to detect anti-proliferative or anti-invasiveness drugs for melanoma (34, 54). A membrane invasion culture system (MICS) was developed using a basement-membrane-like structure to evaluate the ability of a drug to block invasiveness, a desirable characteristic to fight against metastasis. Using the A375 metastatic cell line, its ability to invade Matrigel-filters in MICS chambers was measured after drug exposure by staining and counting cells that remained trapped on the filters. After evaluation of 26 compounds in different dosages, 15 combinations demonstrated more than 60% inhibition of invasion compared to untreated cells. Compounds were previously characterized based on their non-cytotoxic profile in established concentrations through clonogenic assays to ensure that cells would remain viable, i.e., retaining their ability to metastasis (34). A fluorometric assay was developed in the early 90's to screen compounds for anti-proliferative potential based on the ability of cytoplasmatic esterase to metabolize the substrate 4-methylumbelliferyl heptanoate (MUH) in viable cells. The results of this assay correlated with results obtained using the thymidine incorporation method. The test was further validated using cisplatin and vindesine treatments on SK Mel-28 and StML-12 melanoma cell lines (54).

Aiming to improve selectivity, studies from the last decade have explored the potential of photodynamic therapy in melanoma (48, 55–57). Such studies have also employed uptake assays to assess the internalization of photosensitizers by tumor cells. Internalization experiments are commonly based on fluorescent microscopy, confocal microscopy, fluorometry, and spectrophotometry (48, 55, 56). Following preliminary screenings, other *in vitro* techniques are typically utilized to better characterize compounds with promising characteristics for melanoma treatment, including (i) flow cytometry (FC) to detect phosphatidylserine residues that are externalized in apoptotic cells (58) and to assess cell cycle distribution (59); (ii) enzymatic assays to detect caspase activity (60); (iii) TUNEL staining to measure DNA damage (60); and (iv) western blot to detect expression of kinase proteins (60, 61).

Unfortunately, due to the extensive diversity of melanoma tumors and mechanisms involved in cell death processes, it is difficult to elect a single test to predict effective drugs. Moreover, most novel screening approaches described in the literature do not use positive and negative clinical samples nor a single standard treatment as a reference to determine the potential predictive value of new *in vitro* screening techniques, making it difficult to compare the efficacy of these methodologies. Thus, our group has employed a combination of several *in vitro* approaches to determine the potential efficacy of novel targets, including characterization of cell death processes, cell cycle, cytotoxic mechanisms, production of reactive oxygen species, and clonogenic ability (62–66). We believe that the incorporation of multiple techniques results in a more reliable result that could be extrapolated for further *in vivo* tests.

#### Molecular Approaches

Molecular tools have also played an important role in co-localization studies, which are used to evaluate intracellular targets of new anti-tumor compounds. Kleemann et al. employed organelle-targeted GFP and/or YFP-plasmids to characterize location of hypericin in A375, 501mel, and UCT Mel-1 melanoma cell lines (48). Our group has also been working to characterize possible mechanisms of action of BCG-based immunotherapy against metastatic melanoma using reporter recombinant BCG strains (data not published). We have also employed the construction of recombinant BCG strains to serve as potentially stronger immunotherapeutic agents against bladder cancer, with promising results demonstrated through *in vitro* approaches (67, 68). We are currently evaluating the efficacy of this strategy for melanoma treatment.

High-throughput screening based on a gene trap strategies was also developed and validated for malignant melanoma using the A375 cell line harboring a *BRAF* driver mutation (V600E) (69). The approach consists of detecting the inhibition of oncogenic pathways by drugs using a promoterless reporter system that becomes active and emits an “on” signal when integrated in specific loci. Identification of gene traps in relevant oncogenic pathways was performed using known BRAF and MEK inhibitors, vemurafenib and trametinib, respectively. Of the 6000 compounds initially screened, 40 were identified as MAPK pathway inhibitors using this approach.

#### 3D Models

Melanoma progression evolves from a radial growth phase (RGP) to a vertical growth phase (VGP), the stage in which most cases are diagnosed (70). Tumors in the VGP stage are associated with increased metastatic potential and poor prognosis. Moreover, the melanoma microenvironment consists of a network of cells, including fibroblasts, immune cells, endothelial cells, and transformed melanocytes (71). Conventional tests based on detection of enzyme activity do not effectively mimic this complexity. Thus, more complex systems are urgently needed to better understand and mimic the tumor microenvironment, especially for metastatic melanoma, which is often associated with resistance to therapy.

A 3D platform was developed to screen a series of chemotherapeutic drugs using B16-F10 melanoma cell line as a model (72). The platform combines a 3D extracellular matrix with gold electrodes that sense the electrical response after drug exposure. The microfluidic device was able to detect changes in the response of drug-susceptible and drug tolerant B16-F10 cells after carboplatin exposure. Considering that intra- and inter-tumor variability can result in varying levels of chemoresistance for individual tumor cell clones, this kind of approach could emerge as an ideal solution for personalized screening of multiple drugs at once.

Spheroid models have also been developed for evaluation of novel drugs (73). The organotypic 3D model resembles cutaneous melanoma metastasis and represents a more reliable strategy than 2D methods. Vörsmann et al. developed melanoma spheroids of about 500 μm in size using SBCL 2 (RGP stage), WM 115 (VGP stage), and 451-LU (metastatic) cells mixed with collagen I and fibroblasts to form an *in vitro* skin model (74). Using the 3D model, the authors observed an improvement in the therapeutic effect of TRAIL + cisplatin and reduced efficacy of TRAIL + UVB, which is incongruent with what was observed when using a 2D culture system. Three-dimensional models represent a more suitable tool for drug screening for metastatic melanoma. As the metastatic process involves cytoskeletal reorganization and loss of adhesion receptors, destabilizing the cellular interactions with the extracellular matrix, these targets can also be explored to characterize novel anti-melanoma drugs. Changes in cytoskeletal organization have been investigated using fluorescence staining and tubulin polymerization assays; however, this work has been done predominately using 2D models (75). Combining 3D model systems with these evaluations represents a powerful new method for new drug discovery, allowing for investigation of distinct processes contributing to invasion, migration, and metastasis.

Because of these results, we believe it is important to consider the effects of cell-cell interactions in co-culture experiments and the mechanism of action for immune cells when screening potential of new drug targets. Moreover, further investigation of tumor microenvironment is another important factor to be considered when testing therapies against melanoma, especially in the case of metastatic disease. Our group is currently focused on determining the response induced by novel compounds using melanoma cells co-cultured with immune cells, and characterizing pathways activated by these agents.

We also highlight that although *in vitro* assays provide important information regarding the cytotoxic potential of new compounds being screened, they fail to mimic the complex environment of a living animal. Therefore, *in vivo* screening following initial *in vitro* validation of the most promising anti-melanoma compounds represents an essential and rational step in the development and approval of new melanoma drugs, in accordance with the need to reduce, replace and refine animal experimentation and to better select drug candidates. Moreover, the huge tumor variability observed clinically requires the use of a combination of techniques for screening, including the use of different cell lines representative of various genetic, metabolic, and physiologic phenotypes to minimize bias when testing new drugs.

### Section II

#### *In vivo* Models of Melanoma

Animal models are important tools for elucidating the effectiveness of new biomedical compounds and therapies. In fact, in order for a new biomedical product to enter human clinical trials and be approved for commercialization, the treatment must first be tested for efficacy and safety in at least two different animal models (76). Therefore, *in vivo* testing in animal models is a critical step in the screening of new potential compounds with antimelanoma activity. therapies for melanoma.

Since the rise of spontaneous melanoma is extremely rare in animal models, exceptions being three swine lines that develop spontaneous forms of malignant melanoma: the Sinclair miniature white pig (77), the Munich miniature swine troll (78) and the Melanoma bearing Libechov minipigs (MeLiM) (79), it is necessary to induce tumor formation in order to create biological models of melanoma. This induction can be performed using several approaches, including genetic engineering, graft transplantation, and viral/physical/chemical induction. Graft transplantations consist of either xenograft or allograft (syngeneic) depending on whether the donor tumor originates from a different or the same species, respectively (80).

**Figure 2:**
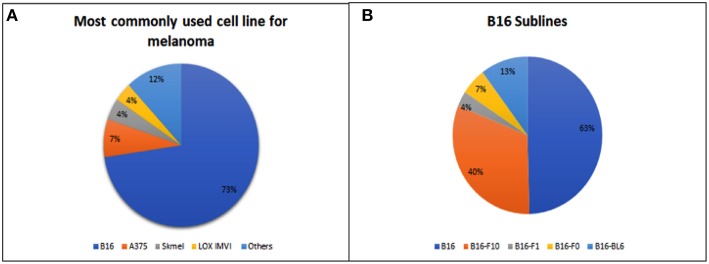
**(A)** Percentage of trials using different cell lines to form grafts in melanoma murine models for *in vivo* therapeutic screening. B16 stands for all B16 sublines. Skmel stands for all Skmel sublines. “Others” include UACC-62, A2058, Na11+, Melanoma xenograft (MEXF), K1735, K1735-M2, HT168-M1, MM96L, Me501, M-14, Me30966, D10, 205, MeWo, VM1, Mel-JD, MEXF 989, WM 266-4, human malignant melanoma (BRO), and M24 cell lines. **(B)** Percentage of trials using each of the B16 sublines in syngeneic tumor models of melanoma for screening *in vivo*. Obs.: B16 indicates articles that do not specify a B16 subline.

The most widely used preclinical model is the murine model. Some of their characteristics, like small size, well-known genetics, easy handling, and inexpensiveness make them the ideal choice for drug screening. Specifically, for melanoma, graft transplantation using B16 murine melanoma cells represent the most widely used animal model. Of the articles published between 1980 and 2018 involving *in vivo* screening of potential antimelanoma molecules, 70% of them used mice bearing B16 grafts for their *in vivo* evaluations (Figure 2A). In this section of the review, we will discuss the most widely used melanoma animal models for *in vivo* screening of drugs and compounds.

#### B16 Syngenic Mouse Model

As mentioned before, the most widely used pre-clinical animal model for melanoma drug screening is the B16 syngenic mouse model, mainly due to its well-known genetics and histological characteristics similar to human melanoma. In addition, because this animal model possess a functional immune system, syngeneic transplantations are frequently used to evalu-ate immunotherapies and interactions between tumor and immune cells (81). Synergistic transplantation of B16 murine melanoma cells into C57BL/6 mice involves inoculation of mice with the same genetic background of the host animal. It is one of the most advantageous experimental models for testing large numbers of drugs in order to select specific compounds for their antimelanoma activity and has been vastly used for this purpose over the past decades. The inoculation can be performed subcutaneously (SC), intraperitoneally (IP), or intravenously (IV) depending on whether the formation of a solid or metastatic tumor is desired.

Several studies use the B16 murine melanoma model to evaluate compounds. Some examples date back from the 80's, where Miura et al. tested the antimelanoma and melanocytotoxic effects of phenolic and catecholic compounds. Among nine compounds tested, 4-S-cysteaminylphenol (CAP) resulted in an increase in the life span of solid melanoma-bearing mice and inhibited the growth of the melanoma tissue (82). In addition the glutamine analog L-glutamic acid y-monohydroxamate (GAH) was tested and proved to considerably increase survival of mice bearing solid B16 melanoma tumors in a schedule-dependent matter (83). Finally, the anti-tumor properties of Cy 233, a new nitrosoureido sugar, was investigated and demonstrated long-term survival of mice bearing B16 solid tumors across all schedules of treatment (84). Together, these studies represent examples of successful *in vivo* evaluation of melanoma treatments using the B16 syngenic mouse model.

More recently, 16K hPRL, a potent inhibitor of angiogenesis was shown to inhibit tumor growth in a subcutaneous B16F10 mouse melanoma model using a gene transfer method based on cationic liposomes (85). TPI-1, a SHP-1-targeted anti-cancer agent, inhibited the growth of B16 melanoma tumors in ~83% of treated mice at a tolerated oral dose in a T cell-dependent manner (86). A systematic study testing MPTQ, a compound containing a novel tetracyclic condensed quinoline ring system, was carried out to evaluate its antitumor efficacy against B16 murine melanoma. In this study, both single and multiple IP doses displayed high levels of activity against the SC grafted B16 melanoma, significantly increasing survival and inhibiting tumor growth (87). Finally, *in vivo* investigation of dipotassium-trioxohydroxytetrafluorotriborate's antitumor effects in a B16-F10 melanoma tumor model demonstrated reduced tumor growth compared to controls (88).

The methodology to obtain and utilize the widely used B16 solid tumor model is well established. The subcutaneous (or sometimes intraperitoneal) injection of about 1 × 10^5^ cells/mouse in C57BL/6J strain mice results in palpable tumor within 5 to 10 days that grow to 1 cm^3^ in 14 to 21 days (89). The results are obtained by comparing the tumor size of the treated groups against the control.

#### B16 Artificial Metastatic Mouse Model

The use of models that mimic invasiveness are important in melanoma drug discovery since melanomas are characterized by their high aggressiveness and ability to metastasize to distant organs (90). Metastatic melanoma is incurable in most cases, presenting a 5-year survival rate lower than 5% (91). Therapeutic options available today for the treatment of advanced melanoma are largely ineffective (5, 92), highlighting the importance of continued improvement in metastatic melanoma treatment through the screening of new compounds for anti-metastatic activity. In this context, IV injection of B16 cells to obtain pulmonary metastases has been used for the past decades in order to investigate the effect of new molecules on metastatic formations. Several studies have performed IV injection of B16-F10 cells into the tail vein of mice, which allows the cells to travel throughout the body and invade other organs, resulting in distant melanoma metastases (93–96).

Using this model, Sharma et al. demonstrated that 7t8OG was able to reduce the number of lung metastases observed in 89–99% of mice harboring B16 metastatic tumors (93). The antimelanoma activity of molecules produced by *Streptomyces griseoluteus* was also evaluated using this approach. One of the tested molecules demonstrated a dose-dependent antimetastatic activity *in vivo*, however, none of them showed activity against solid melanoma tumor models *in vivo* (94). A liposome-based formulation of ET-18-OCH3 was also shown to be more effective in reducing lung tumor nodules in metastatic B16/F10 melanoma bearing mice than non-liposome-based formulations (97).

In more recent studies, B16-F10 tumor-bearing mice with pulmonary metastases were used to screen potential antimelanoma molecules and evaluate their antimetastatic activity, including a specific inhibitor of thrombin, recombinant hirudin with stealthy liposomal vinblastine (98), a heterodimer recombinant (r) IL-7/HGFb that was cloned and expressed as a single-chain hybrid cytokine (95), nanoencapsulated alkanoid Camptothecin (CPT) (99), interferon alpha (100), an aqueous extract from the root of *Platycodon grandiflorum* (96), berberine (101), and RAM, an RGD-non-peptide Analog-Molecule that markedly reduced up to 80% of lung metastases development (102). In addition, the antimetastatic activity of the theophylline analog 7-(2-hydroxyethyl)theophylline (HET) (103), peptides corresponding to conserved complementary determining regions from different immunoglobulins (104), carbamoylphosphonates (CPOs) (105), and C4-benzazole naphthalimide derivatives (106) were also screened using this model.

In addition, the ability of the topoisomerase I inhibitor MONCPT to reduce melanoma metastasis was tested using the B16-F10 metastatic mouse model. However, instead of using the regular B16 cell line, they employed a slightly different approach through the use of a B16-F10 cell line expressing green fluorescent protein (B16-F10-GFP). These cells were also injected subcutaneously in order to evaluate the antitumor effect of the molecule in solid melanoma tumors. The use of B16-F10-GFP recombinant cells allowed the investigators to visualize the resulting tumors using a fluorescent macro-imaging system and fluorescence stereomicroscope. The number of metastatic nodules on the lung surface were counted under fluorescence stereomicroscope to quantify the pulmonary metastases. MONCPT markedly reduced pulmonary metastases in a dose-dependent matter and inhibited tumor growth in the B16-F10 xenograft model (107).

Another alternative approach is to use the melanotic subline B16F10-Nex2, which was developed from B16-F10 cells by the Experimental Oncology Unit (UNONEX) and is characterized by low immunogenicity and moderate virulence. It can form lethal subcutaneous tumors, while pulmonary nodules are formed only when injected IV (108). Three studies have used this subline to produce a lung metastatic melanoma model for screening of potential melanoma treatments. The first evaluated the effect of fastuosain, a cysteine proteinase from *Bromelia fastuosa*. After treatment, very few lung metastatic nodules were detected (108). In another study, FTY720, a compound already approved by the Food and Drug Administration for treatment of patients with multiple sclerosis, was found to limit metastatic melanoma growth (109). Finally, Bechara et al tested the *in vitro* antitumor activity of a Biphosphinic Palladacycle Complex (BPC), followed by *in vivo* studies demonstrating BPC protects mice against metastatic melanoma (110).

The methodology to obtain and utilize B16 artificial metastatic mouse models is also well established. An IV injection of 2 × 10^5^ cells on C57BL/6J mice results in the establishment of visible pulmonary tumor nodules within 3 days. In order to determine the antimetastatic effects of a given drug, the number of lung metastases are counted and compared to a control group.

One big advantage of this model is the extremely rapid formation of lung metastases. However, this is only possible because this model does not mimic the actual events required for metastasis of primary tumors, since the first steps of metastasis (localized invasion and intravasation into the blood vessels) are bypassed when cells are injected directly into the mouse bloodstream (111). This represents the biggest disadvantage when utilizing the B16 artificial metastatic model. To overcome this, B16 sublines with enhanced metastatic ability have been isolated and used to form spontaneous metastases in mice. Despite these limitations, the B16-F10 artificial metastatic mouse model is still a valuable model to test the ability of compounds to inhibit formation of metastatic nodules.

#### B16 Sublines

It is well known that primary malignant tumors consist of a heterogeneous population of cells rather than a homogeneous cellular mass (112). Therefore, it is rational to think that subpopulations within a cell line can present different and unique characteristics from one another. Likewise, the B16 lineage has sublines that present different characteristics. Due to their specific characteristics, each subline is ideal to study different aspects of melanoma. Some sublines, like B16-F1, have a low potential for lung colonization and are useful for studying primary tumor growth (113), while others, for example B16-F10, display a high potential for pulmonary metastasis and are ideal for *in vivo* metastatic studies. The rapid growth and fast development of B16-F10 tumors typically leads to death within 2 to 4 weeks after SC injection into mice (114). Therefore, the ideal subline to use depends on the experimental design and expected activity of the compounds being screened.

There are also some populations of melanoma B16 cells with enhanced metastatic ability. These populations have been identified, selected, and isolated *in vitro* to established sublines characterized by their enhanced invasive properties. One example is the B16–BL6 melanoma cell line, a highly metastatic murine tumor cell. The BL6 variant subline was selected and isolated *in vitro* from B16-F10 cells. It displays greater invasiveness when injected SC or intramuscular (IM) compared to its parental line (B16-F10). However, the variant subline is less efficient than the parent B16-F10 line in producing experimental metastases after IV injection, probably because B16-F10 cells are already highly metastatic when injected IV (115). Similar to its parental cell line, the B16-BL6 can also be used to form solid tumors, and has been used to test novel melanoma treatments, including N-Benzyladriamycin-14-valerate, a novel lipophilic anthracycline with greater *in vivo* antitumor activity than doxorubicin (116), SBF-1, a synthetic steroidal glycoside (117), and surface-charged nanostructured lipid carriers (NLCs) (118).

In addition to B16-BL6, the Mmb16 cell line represents another metastatic B16 subclone (119, 120). It has been used to demonstrate significant antitumoral activity of combination IL-12 and paclitaxel therapy (120), as well as the efficacy of systemic infusion of recombinant human macrophage-colony-stimulating factor in combination with local treatment with human recombinant tumor necrosis factor cx and mouse recombinant interferon (119).

One of the advantages of using B16 sublines with enhanced metastatic ability instead of the parental B16-F10 lines is the ability of these sublines (B16-Bl6 and Mmb16) to form spontaneous lung metastases when inoculated SC or IP. The spontaneous lung metastasis model represents a highly valuable model because B16-Bl6 and Mmb16 cells have to go through all the initial events required for primary tumor metastasis (111, 121), therefore mimicking the metastatic process that occurs clinically. This is in contrast to methods consisting of artificial injection of cells directly into the animal vein. However, it is a much more time-consuming process and therefore not as frequently used.

Other B16 variations include the B16-F0 and B16-F1 cell lines, which are derived from C57Bl/6 mice^2^. But, overall, these sublines are not used as frequently as the B16-F10 cell line (Figure 2B). In fact, B16-F10 is the most widely used of all available sublines for *in vivo* xenograft modeling.

Several characteristics make the B16 an ideal experimental model in drug screening. For instance, it utilizes a well-characterized cell line and tumors are rapidly developed after B16 inoculation. Also, syngeneic transplantations are frequently used to test immunotherapies (81), which represents a major advantage of this model over xenografts because it allows the evaluation of interactions between tumor and immune cells present in the tumor microenvironment. In fact, models with functional immune systems are essential to test immunotherapies that aim to stimulate the body's immune system to target and attack melanoma cells. One example is the melanin-mediated cancer immunotherapy strategy, which consists of transdermal vaccination using a MN patch loaded with B16F10 whole tumor lysate, which resulted in increased survival of C57BL/6J mice (122). The B16-F10 model was also used to test a synergistic immunotherapy strategy targeting both the immunoinhibitory receptor programmed cell death protein 1 and the immunosuppressive enzyme indoleamine 2,3-dioxygenase. Using this model, the antitumor effect of this treatment was demonstrated, including enhanced effective T cell immunity and reduced immunosuppression in the local site (123). In addition, the use of an *in situ* formed immunotherapeutic biorresponsive fibrin gel was able to control local tumor recurrence after surgery as well as the development of distant tumors by ‘awaking' the host innate and adaptive immune systems (124).

However, an obvious disadvantage of B16 syngeneic models is the use of murine cell lines instead of human cells, which have shown to display several differences compared to human melanomas in terms of hallmarks of cancer, including expression of adhesion proteins, growth factor production, and antiapoptotic mechanisms (114). In addition, even though distinct sublines are available, B16 cells were originally isolated from a single inbred mouse strain and therefore do not possess the range of genetic variation observed clinically. Some authors even propose that B16 models should not be used because the data obtained from them can lead to false conclusions. Instead, they recommend using murine models that better recapitulate human melanoma, such as genetically modified mouse models (114).

#### Additional Murine Cell Lines

Although the B16 allogenic tumor model deserves special attention as it is the most widely used melanoma model for *in vivo* drug screening, other murine cells are also available to be inoculated into mice to form graft tumor models. These include K1735 and its subclone K1735-M2, both derived from C3H/HeN mice (86, 94), and the Cloudman S-9 cell line, obtained from the DBA/2 mouse (113, 125), in addition to a variety of other cell lines not addressed here.

These lines have also been used to evaluate new compounds with potential antimelanoma activity. In one study, TPI-1 analogs were tested, with analog TPI-1a4 found to inhibit growth of K1735 melanoma tumors in mice (86). In a screening of actinomycetes for substances with solid antitumor activity, a structure named U-77863 obtained from *Streptomyces griseoluteus* (strain WS6724) exhibited a dose-dependent antimetastatic activity *in vivo* in both K1735-M2 and B16-F10 murine melanoma models (94). Lastly, vitalethine was evaluated in mice inoculated with the uniformly fatal Cloudman S-91 melanoma cell line, displaying substantially diminished tumor sizes as well as increased survival (125). However, these murine cell lines are not as well characterized, and their use for melanoma drug screening is uncommon compared to the B16 cell line.

#### Cell Line Xenograft Models

Xenograft models developed using human cancer cell lines have been widely used in research to answer questions ranging from the efficacy of new therapies to elucidation of the mechanisms underlying tumor biology. This scenario is no different for melanoma, where xenograft models are widely used for the study of metastases and drug screening (126). Consistent with the methodologies employed to obtain solid tumors in syngeneic models, an amount of ± 2 × 10^6^ human cells are injected usually SC into immunodeficient mice to generate xenograft models of human melanoma.

The biggest difference in injecting either mouse or human cells into mice in order to form grafts is that human cells lines need to be injected into immunosuppressed or imunocompromised mice to avoid rejection by the host immune system. These models are called xenograft models, meaning that the tumor donor and the host animal belong to different species. Severe compromised imunodeficience (SCID) and athymic nude mice are the most commonly used animals for this purpose. They both lack an efficient immune system, limiting the ability of these mice to recognize and reject the human cells, allowing the injected human cells to grow in the mouse model and form tumors. More specifically, nude athymic (nu/nu) mice are T-cell deficient (127) and SCID mice are both T and B-cells deficient (128).

A375 is one of the most commonly used human cell lines for mouse xenograft development and subsequent compound screening. In nude mice bearing well-developed human A375 melanoma xenografts, administration of 125I-labeled ZME and ZME-gelonin was tested for its antimelanoma activity in solid and metastatic tumor models. The results showed suppression of tumor growth and a 213% increase in mean survival time in the immunotoxin group compared to the control group (129). Other compounds, including 4-substituted methoxybenzoyl-aryl-thiazoles (SMART) (130), YM-201627 (131), and the organopalladium compound tris (dibenzylideneacetone) dipalladium (Tris DBA) (132) were tested using A375 melanoma xenografts. Other generally used human cell lines include the Skmel sublines and LOX human amelanotic melanoma cell line.

Although used frequently for compound screening, cell line xenograft models are poorly pre-dictive of clinical outcomes, as evidence by the high proportion of drugs demonstrating efficacy in these models that ultimately fail in clinical trials (133). This is mainly due to establishment of melanoma cell lines under artificial conditions during cell culture growth and *in vivo* passaging over several years resulting in selection of clones that are no longer representative of the original tumor (80). To overcome this, the use of primary mela-noma cells for xenograft development has also been reported and will be addressed in the next section of the review.

In addition, these models do not possess a functional immune system, which precludes their use in immunotherapy studies. Despite these disadvantages, xenografts are widely used due to the ability to produce tumors using human melanoma cells. In mice, these melanoma cells can interact with the bloodstream, lymphatic vessels, and tissue stroma, providing valuable information regarding compound effectiveness in this context. (134).

As discussed in this section, all *in vivo* graft models have advantages and disadvantages. Therefore, to obtain results translatable to preclinical and clinical trials, we believe that compounds should be tested simultaneously in more than one murine model. The data obtained in each model (syngeneic and xenograft) would complement each other, since the advantages of one model usually represent disadvantages of another. In addition, the use of genetically modified mouse models that better reflect the human disease could help address questions not possible using graft models.

#### New Models

In order to improve the process of screening compounds with potential antimelanoma activity, a number of research groups have worked to develop new models with various pros and cons, which are discussed in depth below.

#### Patient-Derived Tumor Xenograft (PDTX)

Several studies have demonstrated that PDTX models are more suitable for mimicking human tumors than traditional cell line xenografts (135, 136). The goal of PDTX models is to promote personalized medicine by allowing the animal model to bear human tumor cells originated from actual patients. This platform allows scientists to test and evaluate efficacy of drugs and therapies on the patient's own cells. PDTX models have led to discovery of drug resistance mechanisms common found in metastatic melanoma patients and are also useful for identifying combination therapy regimens that could prevent drug resistance (135). PDTX models are also heterogeneous in nature and therefore more closely reflect tumors observed clinically.

To develop PDTX models, tumor cells are obtained from a surgically resected clinical tumor sample. The tumor mass serves as the raw material from which small specimens are obtained. These specimens are then transplanted SC into immunodeficient mice to produce tumors derived from the patient's malignant cells (136). However, this is a time consuming and technically challenging technique, with palpable tumors developing between 3 to 9 months, with many mice failing to develop tumors, which represents its biggest disadvantage of this model (135, 136).

Although this approach allows for more personalized drug discovery and has a huge potential in melanoma drug screening, it hasn't been widely employed to date. One example using PDTX models for drug screening is the work of Hollingshead et al. describing the preclinical basis for further development of 17-dimethyl aminoethylamino-17-demethoxygeldanamycin hydrochloride (17-DMAG, NSC 707545). In this work, four melanoma xenografts (MEXF 276, MEXF 462, MEXF 514, and MEXF 989) were derived from clinical surgical specimens and directly implanted into nude mice aiming to perform the *in vivo* efficacy studies (137).

#### Genetic Engineered Mouse Models (GEMM) of Melanoma

Cancer is a multifactorial diseases trigged by genetic perturbations in genes related mostly to cell proliferation, cell cycle, and apoptosis (138). In this context, elucidating the genetic underlying melanoma development is an essential step to fully understand the disease and improve melanoma treatment. To this end, genetically engi-neered mouse models (GEMMs) have been vastly used to investigate the effect of genetic alterations in the processes of melanoma initia-tion, progression, and metastasis (139).

GEMMs are mostly used to unravel the molecular mechanisms related to melanoma development and drug resistance rather than in the drug screening process itself. However, they have been very useful for elucidating gene function and identifying key targets for therapeutics. Examples of genetic engineering models (GEMs) for melanoma are showed in Table 3.

**Table 3:** Examples of GEM for melanoma.

**Animal**	**Gene modified**	**Function/goal**	**References**
Zebrafish	*BRAF^*V*600*E*^*; *p53*-deficient	Study the genetic basis of melanoma initiation and development,	(140)
Zebrafish	*NRAS^*Q*61*K*^*; *p53*-deficient	Study the genetic basis of melanoma pathogenesis	(141)
Zebrafih	*HRAS^*G*12*V*^*	Study the molecular basis of melanoma formation and progression	(142)
Zebrafish	*HRAS^*G*12*V*^*	Provide a link between kita expressing melanocyte progenitors and melanoma and offer the advantage of a larval phenotype suitable for large scale drug and genetic modifier screen	(143)
Zebrafish	*GNAQ*^*Q*209*P*^; *p53*-deficient	Study the correlation between oncogenic GNAQQ209P mutation and sustained ERK1/2-MAPK activation	(144)
Mouse	*HRAS^*G*12*V*^*; *p53*-deficient	Study the genetic basis of melanomagenesis	(145)
Mouse	*NRAS^*Q*61*K*^*; *INK4a*-deficient	Obtain a novel mouse model with melanotic and metastasizing melanoma	(146)
Mouse	*BRAF^*V*600*E*^, INK4A/Arf-deficient*	Produce a pre-clinical model of mutant BRAF function in melanoma	(147)
Mouse	*HGF/SF-Tg*	Study the genetic basis of melanoma formation and progression	(148)
Mouse	*BRAF^CA^;* *Cdkn2a^lox/lox^*; *PTEN^lox/lox^*	Study the mechanisms driving melanoma metastasis	(149)

#### Zebrafish and Porcine Models

Another model that is of interest for melanoma drug screening is the zebrafish (*Danio rerio*) embryonic model because it allows the investigation of antitumor drug properties within 1 week, in addition to being suitable for toxicity screenings. The optical transparency of the zebrafish also provides the unique opportunity to monitor fluorescently labeled cancer cell growth over time. Interest in this model has been growing due to its rapid development, low cost, and minimal amounts of compounds and housing requirements (150). Added to this, similarities between human and zebrafish larvae in terms of genetics and the physiology of the innate immune system makes this model ideal for melanoma studies (151). Despite this, the model does have some limitations, including the route of compound delivery (dissolving the compound in egg water, diffusion through the skin and gills, or absorption via the gastrointestinal tract), and whether these compounds pass through the blood-brain barrier needs to be clarified (152). Some groups have started to investigate some of these concerns, as Fleming et al. who used fluorescent labels and capture compounds to assess blood-brain barrier permeability (153–155).

Zebrafish models can also be genetically engineered to closely recapitulate the genetic background and characteristics of human melanomas. In the same way that GEMMs are usually employed, genetic engineered zebrafish models are most commonly use to elucidate the genetic basis of melanoma initiation, development, and progression rather than for drug screening. Examples of genetically modified zebrafish models for melanoma are provided in Table 3. However, there are examples of zebrafish genetic modified models that were first used to model genetic characteristics of melanoma (142) and later employed for *in vivo* validation of targeted melanoma treatments (156).

One more extremely relevant animal model is the swine. Swine are known to hold great resemblance toward humans in several aspects, including genetic, physiologic, and anatomic levels. A review from Bourneuf et al. discusses the advantages of using swine models to study the genetic basis of spontaneous melanoma, specially the MeLim minipig. He states that swine are an important model for studying spontaneous melanoma development because they recapitulate features of human melanoma, and that a spontaneous porcine melanoma model could be extremelly valuable for investigating melanoma genetics. In addition to the above-mentioned similarities with humans, the location of the melanocytes is the same in both species, being found in the basal layer of the epidermis. This is in contrast to mice where the melanocytes are located in the dermis. As such, pig skin is expected to better reflect healthy and neoplastic human tissue (157).

The knowledge of the pig's genome sequence, which shows great similarity with humans (158, 159), combined with the advancement in genetic engineering techniques makes genetic engineering a powerful tool for developing transgenic porcine models for cancer drug discovery. These platforms represent a more robust model than swine that develop spontaneous melanomas because they can be engineered to express mutated genes frequently found in human tumor, allowing for generation of personalized models that closely mimics the human disease.

To this end, our group in partnership with collaborators has developed the genetically modified Oncopig cancer model, a transgenic pig harboring Cre recombinase inducible transgenes representing two of the most common genetic mutations found in human cancers (*TP53*^*R167H*^ and *KRA*S^*G12D*^) (160). This genetically defined porcine cancer model holds the potential for generating several types of cancer, including melanoma. In a review published by Segatto et al. pigs were proposed as a complementary model for phenotypic drug discovery (PDD) of new cancer therapies due to their metabolic, physiological, and genetic similarities with humans (161).

### Section III

#### *In silico* Drug Assays for Melanoma

The use of alternative methodologies for the development of new compounds with potential antimelanoma activity has rarely been applied in recent years to complement currently used *in vitro* and *in vivo* approaches. These approaches have been developed in order to minimize the use of laboratory animals for experimental testing, as well as to provide additional safety evaluations for subsequent preclinical tests (162). Thus, the use of *in silico* methodologies, such as molecular docking, addresses the need for reduction, replacement, and refinement of animal use in research (3Rs). As we can see in Table 1, the drugs currently used for the treatment of melanoma did not undergo *in silico* testing as part of their development process. The inclusion of such tests represents a more rational approach to screening that can help reduce both the number of animals required and the time and money invested in each molecule. While it is well known that *in vivo* testing is still essential, there are ways to incorporate alternative processes prior to animal testing so that only promising compounds are advanced to animal studies. In addition, using docking studies that predict interactions between molecules and their receptors save time and money during the costly process of discovery and development of new drugs (163).

Researchers have focused on developing numerous software's to aid in this initial developmental process (164). Using these approaches, millions of molecules can be tested computationally to investigate the effectiveness of structure-activity relationships (binding affinity, prediction of the binder's conformation/orientation) and identify cell types it is likely to be most effective in Thiel and Hummer (165), Meng et al. (166). These “*in silico*” approaches therefore allow researchers to choose the best candidates to advance to *in vitro* and *in vivo* testing.

**Figure 3:**
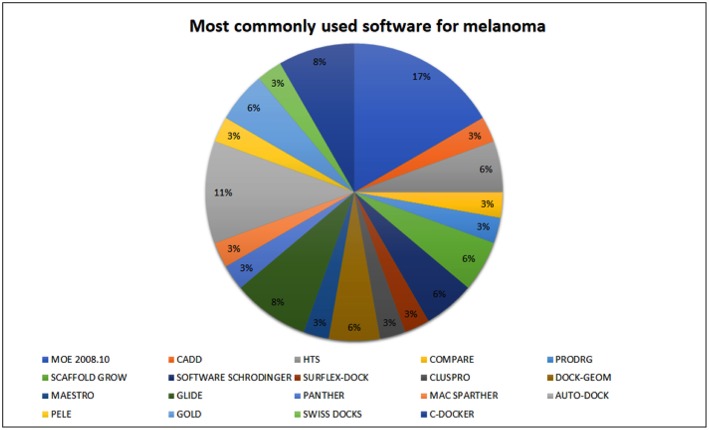
Software most commonly used for melanoma drug screening. The most commonly used software is MOE (17%), followed by HTS (11%).

A literature search was performed to identify programs most frequently used for the development of compounds with potential antimelanoma activity over the past few decades (Figure 3). In this review, we will focus on MOE, HTS, GLIDE, and AutoDock. The Molecular Operating Environment (MOE) is one of the most widely used programs availablely by the research community. This software was developed and is available for purchase through the Chemical Computing Group (CCG). However, there are teaching licenses available on the group's website. This program is capable of performing many functions, including structure and fragment-based design, pharmacophore discovery, biological and medicinal chemistry applications, molecular modeling and simulations, and protein and antibody modeling, among others^3^. Ismail et al. (167) used this program to sharpen the joints of topoisomerase II DNA gyrase (167). In addition, Al-Suwaidan et al. used this feature to verify whether their ligands were formed at the proposed receptors (EGFR-TK ATP binding site) (168, 169). Furthermore, Hassan et al. used this software to identify new candidates for cancer treatments with higher efficacy and lower toxicity, identifying the possible mechanisms of 13a binding in the CDK2 enzyme (170).

High throughput screening (HTS) is the second most widely used software for screening for melanoma treatments. It is a scientific experimental method that selects large libraries of compounds for activity against biological targets through automation, miniature assays, and even analyzes the data on a large scale. With the aid of HTS it is possible to quickly identify active compounds, antibodies, or genes that modulate a specific biomolecular pathway, providing starting points for drug design and an understanding of their role in biochemical processes (171). However, access to this software requires a high investment. To run the HTS requires a highly specialized screening laboratory, so in many cases, due to the high investment costs associated with this *in silico* tool, small to medium research institutions typically use the services of an existing HTS facility instead of developing one themselves. These facilities are typically run by companies, but some universities also have HTS facilities. Hwang et al. used the HTS tool to selectively activate p53 and inhibit NF-κB at the same time, as a strategy for anticancer activity (172). Zimmer et al. also used HTS to induce molecular inhibitors of the S100B protein for melanoma therapy (173). Although an interesting technology, there are some limitations, including a high number of false-positives. Because of this, virtual screening (VS), a totally *in silico* method, emerged as an alternative to HTS (174–176).

GLIDE and Auto-Dock are two other commonly used programs. GLIDE presents itself as complete software for determining anchoring between receptors and ligands. With the help of this tool, productivity increases considerably and the costs of drug development are reduced. What makes this tool even more interesting is that it has a high throughput virtual screening mode (HTVS) included in its system, as well as a standard precision (SP) mode so you can reliably anchor hundreds of thousands of libraries. Another important feature is its ability to reduce false positives using the “false-positive extra precision (XP)” mode. This software is also available through academic licensing^4^. Wang et al. (55) used this tool to identify and obtain molecular structures of potential inhibitors of B-Raf ^V600E.^ (177). Quirit et al. (178) aiming to inhibit the proliferation of human melanoma cells, performed *in silico* binding simulations with the crystallographic structure of NEDD4-1, showing that each of the indolecarbinol compounds bound to the catalytic HECT domain purified from NEDD4-1 (178).

AutoDock 4.0 is a free to download software featuring a set of automated docking tools. It is designed to predict how binding of small molecules, such as substrates or drug candidates, occurs to a receptor of known 3D structure. Easy access to this free software has stimulated its use by academic research groups, where basic and initial research is usually developed. AutoDock has already been distributed to more than 29,000 users worldwide. Among the advantages cited by the creators of the software include its speed and ability to provides high quality predictions and correlations between predicted and experimental inhibition constants^5^. Luo et al. (179) and Ruan et al. (59) used AutoDock to evaluate the antiproliferative activity of melanoma cell lines, in order to run a coupling simulation to insert a compound of interest into the crystal structure of tubulin to determine the likely binding pattern (41, 42). While it is clear that some programs are more complete than others, the cost to research groups must also be taken into consideration. In this context, AutoDock software is a very interesting tool for researchers focused on the synthesis of new compounds.

*In silico* tools are undoubtedly of great value for the initial steps of drug screening. With the aid of these tools, thousands of compounds can be tested to effectively identify candidates for *in vitro* and *in vivo* testing while considering multiple endpoints during a single assessment. Thus, the inserted models can evaluate multiple effects, providing a more comprehensive prediction. However, these approaches, like all techniques, have limitations such as the high cost of commercially available software, the need for high performance processors, the high number of false-positives predicted by software like HTS, and additional uncertainties due to the absence of toxicological data. Although there are limitations, research groups have developed strategies to lessen their impact. There is no doubt that *in vitro* and *in vivo* testing is essential for drug development. Nothing thus far replaces pharmacokinetic and pharmacodynamic tests with such precision as that of a living organism. However, the available systems are based on validated models and well-established REA and QSTR information, which has tended to rationalize the testing, acceptance, recommendation and inclusion of *in silico* methods in several organizations around the world such as European Community, United States Environmental Protection Agency (USEPA) and Food and Drug Administration (FDA). Because of this, we believe that the results of *in silico* methodologies tends to make subsequent tests more effective and predictable and are essential for the screening of new molecules.

## Perspectives and Conclusions

Several approaches are available for melanoma drug screening, including *in silico, in vitro*, and *in vivo* methods, even though few studies have explored the union of these methodologies. *In silico* techniques represent a necessary first step in the screening process and a potential predictive test with the ability to evaluate thousands of molecules and identify the 5–10 with a greater chance of success. In addition to being able to better identify drug candidates, it is possible to exploit drug repositioning, which is a cheap and safe strategy for researchers. In the future, it would be ideal if these computational simulations could be applied more comprehensively using a single software that would simultaneously provide information on cell lines, proteins, and receptors.

The need to understand and mimic the tumor microenvironment *in vitro* has promoted the development of 3D culture models, aiming to reduce the limitations of other *in vitro* tests. We believe that the union of molecular docking with *in vitro* models, such as 3D cultivation, will provide more direct and reliable results. In the period analyzed by our group, few studies used the triad of tests that we consider essential, which demonstrates the need to evolve our future drug screening process in this direction.

Regarding *in vivo* screening, not much has changed regarding the xenograft models used for melanoma drug screening over the past four decades. Although other robust animal models have been developed recently, the “go to” graft model for *in vivo* screening of antimelanoma compounds continues to be the B16 mouse model, even though it represents an unsatisfactory model. Nowadays, *in vivo* drug screening is also performed using additional robust tools to test the efficiency of new molecules and therapies, such as human cell line xenograft models, patient-derived-xenograft models, zebra fish, and GEMs.

We conclude that in order to obtain reliable data when screening potential antimelanoma compounds, researchers should explore several of the currently available animal models options, since (i) one single model is not able to answers all the essential questions required for refined and reliable drug screening, and (ii) one approach usually complements the other. Therefore, by choosing the most promising compounds through initial *in silico* and *in vitro* approaches, it is possible to optimize and reduce animal use by testing a smaller number of potential compounds in multiple *in vivo* models in parallel.

**Figure 4:**
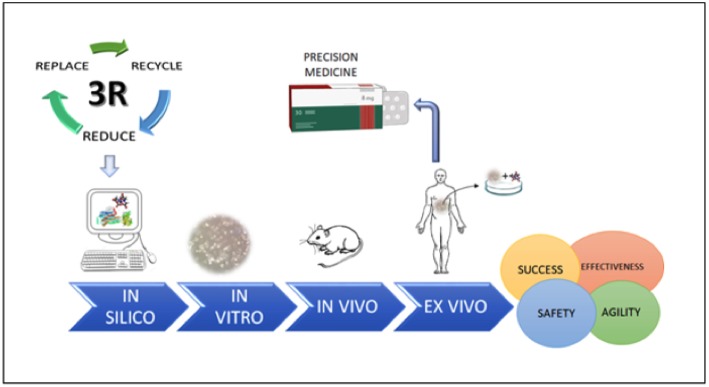
The steps necessary for safe, agile, and effective drug screening, which represent important steps for future development of precision medicine.

In conclusion, we highlight that rational drug screening should respect the sequence of *in silico*/*in vitro*/*in vivo* testing, which will provide more promising drug candidates supported by robust data for preclinical trials, minimizing the unnecessary use of laboratory animals with regards to the 3R's. This sequence is of fundamental importance as we move toward an era of precise personalized medicine (Figure 4).

## Author Contributions

GC, NS, TO, FS, KS, and TC had an equal participation in writing and approving the present manuscript.

### Conflict of Interest Statement

The authors declare that the research was conducted in the absence of any commercial or financial relationships that could be construed as a potential conflict of interest.

## Footnotes

^1^https://www.fda.gov/patients/drug-development-process/step-5-fda-post-market-drug-safety-monitoring

^2^Information extracted from: https://www.atcc.org

^3^Information extracted from: https://www.chemcomp.com/MOE-Molecular_Operating_Environment.htm

^4^Information extracted from: https://www.schrodinger.com/glide

^5^Information extracted from: http://autodock.scripps.edu/
